# Occurrence and management of two emerging soil-dwelling pests ravaging cabbage and onions in Kenya

**DOI:** 10.1038/s41598-023-46190-0

**Published:** 2023-11-03

**Authors:** Lawrence O. Ouma, James W. Muthomi, John W. Kimenju, Dennis Beesigamukama, Sevgan Subramanian, Fathiya M. Khamis, Chrysantus M. Tanga

**Affiliations:** 1 International Centre of Insect Physiology and Ecology, P.O. Box 30772-00100, Nairobi, Kenya; 2 Department of Plant Science and Crop Protection, University of Nairobi, P. O. Box 29053 – 00625, Kangemi, Kenya

**Keywords:** Ecology, Environmental sciences

## Abstract

Cabbage and Onion production in sub-Saharan Africa face numerous pest constraints that needs to be overcome to feed the rapidly growing population. This study aimed to establish the occurrence, incidence, and severity of soil-dwelling pests of cabbage and onions, and current management practices in five Counties of Kenya. Our findings revealed that most farmers grew hybrid vegetables on a small scale, which were highly dominated by various pest species (*Delia platura, Maladera* sp., and *Agriotes* sp. for cabbage and *Atherigona orientalis* and *Urophorus humeralis* for onion. The occurrence, incidence and severity of the various pest species on both crops varied considerably. Over 95% of the farmers relied on synthetic insecticides, which were applied weekly or bimonthly with limited success. Our findings demonstrate that invasive and polyphagous *A. orientalis* and *D. platura* were the most devastating pests of onion and cabbage without effective control options. Therefore, effective, sustainable, and affordable management strategies are required to control the spread of these pests to other crops in the region.

## Introduction

Over the past four decades, food imports have tripled in Sub-Saharan Africa (SSA)^1^, partly due to crop pests and diseases^2,3^. This unfortunate trend has plunged a significant fraction of the SSA population into hunger, food insecurity and extreme poverty^4^. Horticulture is the third largest foreign exchange earner in Kenya, creating over 400,000 direct jobs and supporting over six million livelihoods^5,6^. Cabbage and onions are the most commonly grown vegetables for dietary and economic functions^7^. However, the yields of the two crops have declined due to pest infestations arising from climate change and soil degradation^2,8,9^. Consequently, about half of the onions consumed in Kenya are imported from neighbouring countries (Tanzania and Egypt) and overseas (India)^7^.

The most documented insect pests of cabbage include the diamondback moth (DBM) (*Plutella xylostella* Linnaeus), aphids (*Brevicoryne brassicae* Linnaeus), cabbage white flies (*Aleyrodes proletella* Linnaeus) and Serpentine leaf miners (*Liriomyza brassicae* Riley)^10–12^. Thrips (*Thrips tabaci* Lindeman), Leek Moths (*Acrolepiopsis assectella* Zeller), Onion maggots (*Delia antiqua* Meigen) and Aster leafhoppers (*Macrosteles quadrilineatus* Forbes) are the key onion pests^13–16^. Soil degradation and climate change have caused the proliferation of soil-dwelling insect pests, which have threatened vegetable production^2,17^. The detrimental stages of these pests are found in the soil or within the crop, below or at ground level^2,18^. Some of the noxious soil-dwelling insect pests of cabbage include cabbage root flies (*Delia* spp*.*), white grubs (*Maladera* sp*.*), and wireworms (*Agriotes* sp.)^19–21^. Onion maggots (*Delia* spp*.*) and seedcorn maggots (*Delia* spp.) are undoubtedly the most devastating root maggots of onions^22–25^. *Delia platura* Meigen and *Atherigona orientalis* Schiner have been reported as major pests of crops in the Brassicaceae, Poaceae, Alliaceae, Fabaceae, and Cucurbitaceae families; however, the latter is sporadically reported as a minor pest of onions in countries, such as the United States. Nocturnal insect pests such as black cutworms (*Agrotis ipsilon* Hufnagel) forage at night and tunnel into the soil near the host plant to hide during the day. This behavior makes such pests difficult to control, yet they cause huge crop losses^12,26,27^.

Management of soil-dwelling insects has been difficult due to their abstruse nature. Initially, organochlorines were used to control soil-borne pests, but such chemicals have been banned due to their harmful effects on environmental and human health. These chemicals are applied excessively when targeting soil-borne pests compared to above ground insect pests^2,28^. Additionally, soil-borne insect pests are able to deploy numerous defence mechanisms, ranging from the release of pathogen alarm behaviour to mutual grooming habits to get rid of conidia from the cuticles^29,30^. Others, such as white grubs and cutworms, forage at night and hide in the soil during the day^27,31,32^. Consequently, farmers tend to apply excessive doses of broad-spectrum pesticides, which have culminated in pesticide resistance and pest resurgences. Effective monitoring of soil-borne pests would generate the information necessary for designing efficient control measures.

Although soil-borne insect pests are significantly detrimental to vegetable farming, they are generally poorly documented in SSA. This can be attributed to their cryptic feeding habits, which complicate scouting and monitoring practices^33^. Monitoring soil borne insect pests is also complicated by other factors associated with their life cycle. For instance, species in the Scarabaeidae family (scarabs or beetles) complete their life cycle in approximately one year^14,34^, while the termite life cycle varies from 3 to 8 years, during which they cause critical crop damage of up to 100%^34,35^. Furthermore, soil degradation, habitat destruction, and climate change have increased the proliferation of soil-borne pests^2^.

There is limited information about the incidence, and management practises of soil-borne pests in key horticultural crops in Kenya. The lack of well-defined management recommendations and accurate information on the ecology, lifecycle, and seasonal phenology of soil-borne pests has undermined sustainable management. Although cultural management practices have proven effective in reducing incidences and severity of aboveground pests^36^, there is limited information about their efficacy in the management of soil-dwelling insect pests. The current study sought to ascertain the occurrence, incidence, and severity of soil-borne insect pests of cabbage and onion and their management approaches in various agro-ecological zones of Kenya, to generate information for the development of appropriate control measures.

## Results

### Challenges faced by vegetable farmers

**Figure 1:**
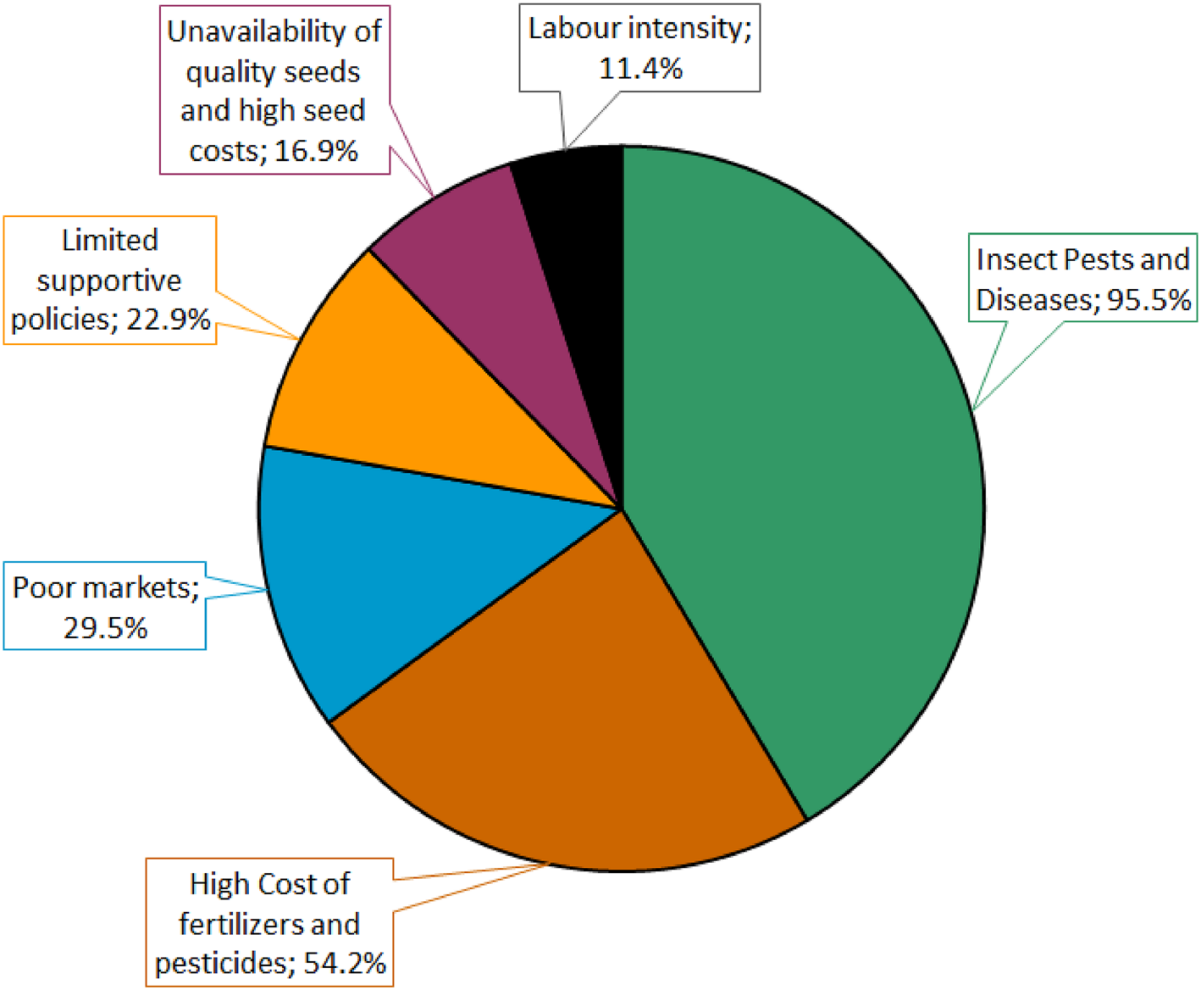
Major constraints to vegetable farming in the study area.

Crop pests and diseases were mentioned as the most critical challenges affecting vegetable production in the study area. The high cost of agricultural inputs, especially pesticides and fertilisers, was also mentioned by a large fraction of the farmers. Several farmers located away from tarmac roads and urban markets cited poor market prices as a key challenge to vegetable farming (Fig. 1). Nearly a quarter of the sampled farmers were concerned over the lack of supportive government policies for upscaling vegetable farming. The challenges of low quality, costly and scarce seeds, and expensive labour were also cited by a considerable number of farmers (Fig. 1).

### Occurrence, incidence and severity of soil-dwelling insect pests

**Figure 2:**
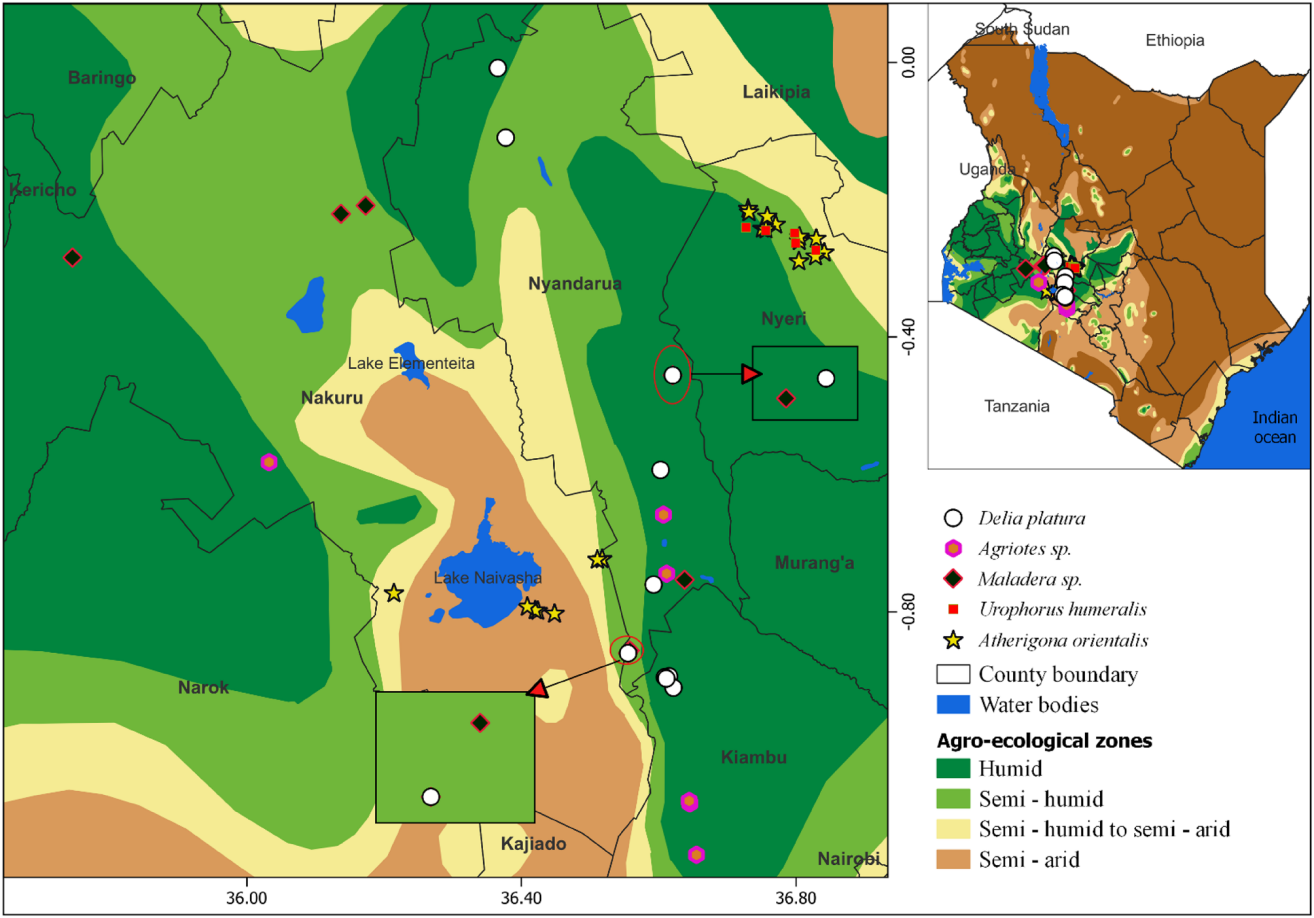
Distribution of soil-dwelling insect pests across various agro-ecological zones of Central Kenya.

The study revealed five typical soil-borne pests in major vegetable growing areas. These were: cabbage maggots/cabbage root flies (*Delia platura*), white grubs (*Maladera* sp.), click beetles (wireworms) (*Agriotes* sp.), onion maggots/onion root flies (*Atherigona orientalis*), and sap beetles (*Urophorus humeralis* Fabricius) (Fig. 2). Cabbage maggots had the highest occurrence in the cabbage-growing upper highland AEZs (UH1) in Nyandarua and Kiambu. White grubs and wireworms occurred most in upper highland AEZs (UH1 and UH2) in Nyandarua, Nakuru, and Kiambu (Fig. 2). However, onion maggots were prevalent in both lower and upper highland AEZs (UH2, LH3, and LH4) in Nakuru and Nyeri. Sap beetles were also identified on mature bulb onions from Nyeri (UH2). The influence of AEZ on pest occurrence was statistically significant (χ2 = 41.13, df = 20, *p* = 0.004). Despite being a central onion-growing zone, the study did not reveal any soil-borne insect pests of economic importance in Kajiado.

**Figure 3:**
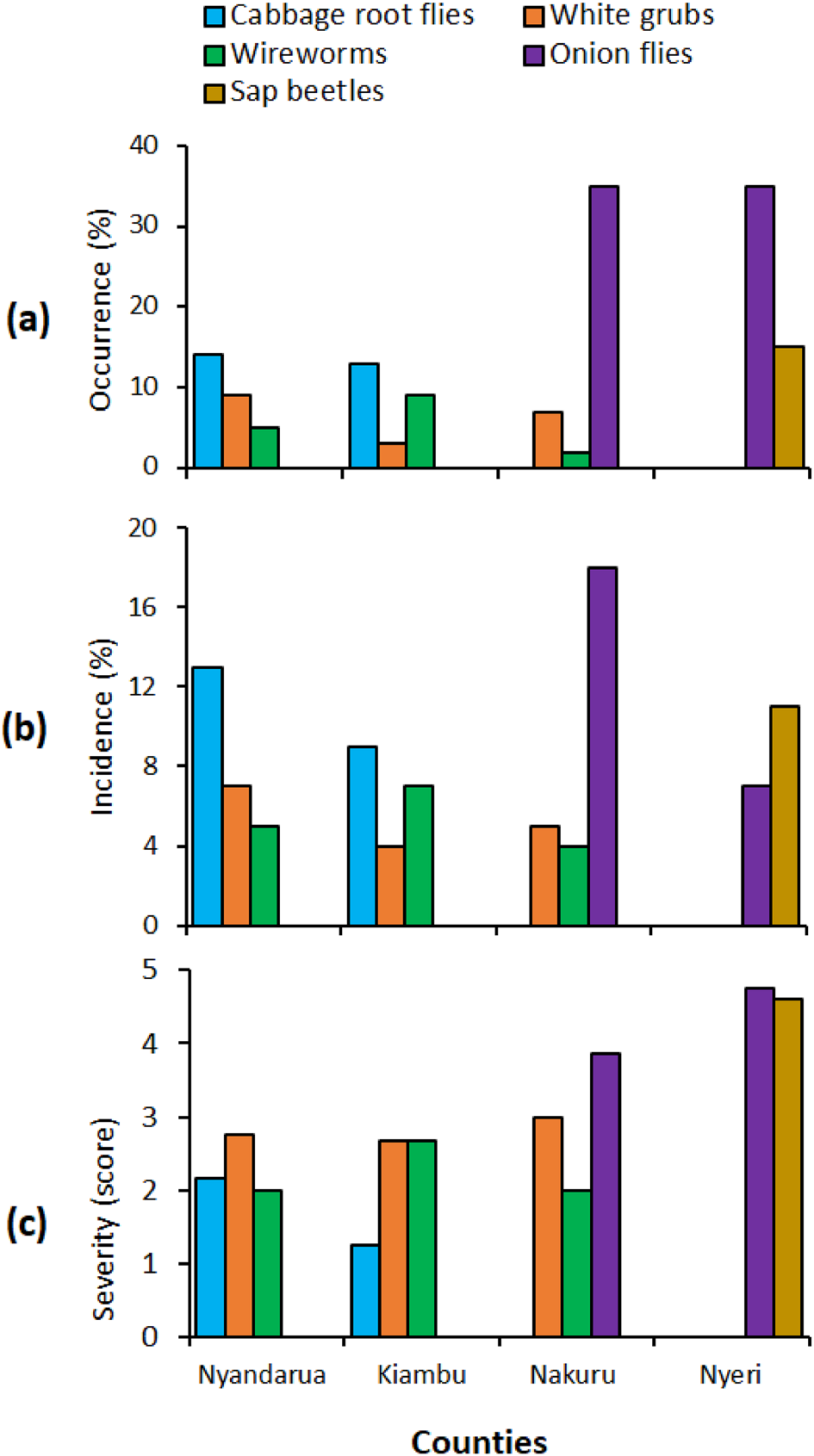
Occurrence (**a**), incidence (**b**), and severity (**c**) of soil dwelling insect pests in the five counties.

*Delia platura* had a significantly higher incidence in cabbage farms in Nyandarua compared to Kiambu (*t* = 1.89, df = 8, *p* = 0.041). *Maladera* sp. and *Agriotes* sp. had a relatively low incidence in Nyandarua, Nakuru, and Kiambu, with a statistically similar occurrence across the three counties (*t* = 0.10, df = 11, *p* = 0.92) (Fig. 3). *A. orientalis* had a significantly higher incidence in Nakuru than in Nyeri (*t* = 3.22, df = 8, *p* = 0.01), whereas sap beetles were only detected in Nyeri (Fig. 3). The incidence of sap beetles and onion flies in Nyeri was statistically similar (*t* = 2.13, df = 15, *p* = 0.296).

In cabbage fields, the damage index ranged between 1.3 and 3.0. White grubs had a higher damage index in Nyandarua, Kiambu, and Nakuru compared to wireworms, but the difference was not statistically significant (*t* = 2.23, df = 10, *p* = 0.149) (Fig. 3).

*Delia platura* had a moderate damage index with higher severity of infestation in Nyandarua compared to Kiambu (*t* = 2.45, df = 6, *p* = 0.022). High damage indices were observed in onion fields; *A. orientalis* and *U. humeralis* had a significantly higher damage index in Nyeri than in Nakuru (*t* = 2.31, df = 8, *p* = 0.04). It was noted that the damage due to *U. humeralis* infestation occurred in association with fusarium basal rot, which caused severe wilting and death of plants. The damage indices of sap beetles and onion flies in Nyeri did not vary significantly (*t* = 2.45, df = 6, *p* = 0.608).

### Factors influencing the incidence and severity of soil-dwelling pests

The education level of farmers was weak positive correlation with the incidence and severity of soil-borne pests in onions and cabbage (Table 1). Agroecological zones were moderately correlated with pest incidence, while altitude had medium and significant negative relationships with pest incidence and severity, respectively. Nevertheless, the mono-cropping system had a weak positive but significant relationship with the damage index compared to a mixed-cropping system that exhibited a non-significant relationship. The level of farmers’ awareness of soil-borne pests had a significant and medium negative relationship with both pest incidence and damage index. The frequency of pesticide application had weak correlation with pest incidence and severity. Furthermore, the pest incidence and damage index had a weak but significant negative correlation with crop yield (Table 1). The study revealed a moderate positive correlation between cabbage variety and pest incidence, and a weak positive correlation between onion varieties and pest incidence (Table 1). Crop yield had a medium and significant negative correlation with pest incidence and severity.

**Table 1:** Correlation between socio-economic factors and the incidence and damage severity of soil-borne pests.

Explanatory variables	Response variables	
	Pest incidence	Damage severity
Education level	0.17	0.03
Agro-ecological zone (AEZ)	0.46**	0.20
Altitude	− 0.35*	− 0.56**
Growth stage	0.05	− 0.12
Cropping system	0.24	0.32*
Awareness of soil-borne pests	− 0.32*	− 0.49**
Frequency of pesticide application	− 0.10	0.02
Onion variety	0.18	0.28
Cabbage variety	0.39*	0.52**
Crop yield	− 0.32*	− 0.44**

** and *Denote statistical significance at *p* < 0.01 and *p* < 0.05, respectively.

### Morphological characteristics of the collected insects

**Figure 4:**
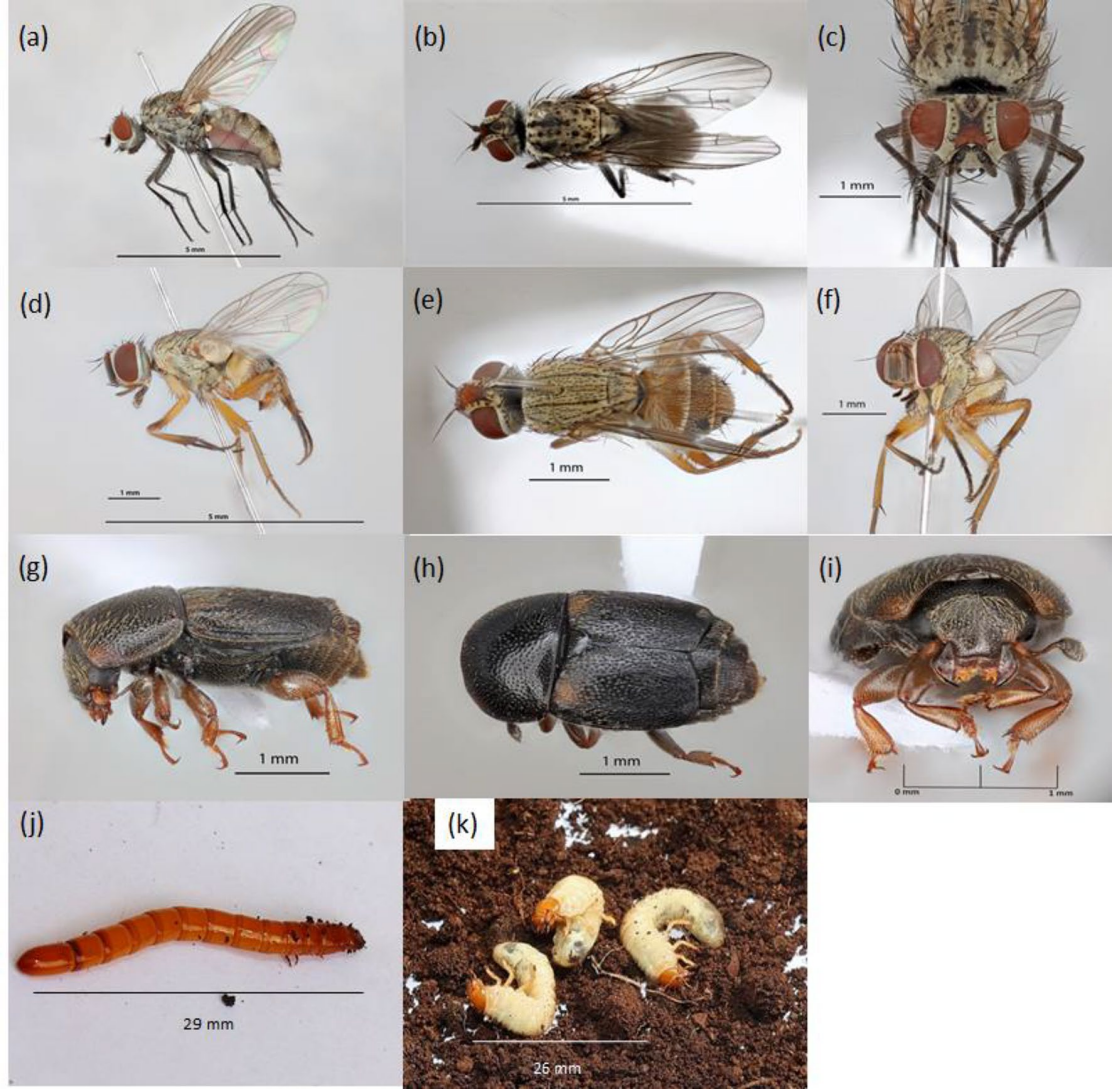
Soil-dwelling insect pests identified in major vegetable growing areas of Kenya. *Delia platura*: ventral (**a**), dorsal (**b**), and anterior views (**c**); *Atherigona orientalis*: ventral (**d**), dorsal (**e**) and anterior views (**f**); *Urophorus sp*.: ventral (**g**), dorsal (**h**) and anterior views (**i**); *Agriotes* sp. (**j**), and *Maladera* sp. (**k**).

Cabbage root fly (*Delia platura*), have a greyish body about 5 mm long, no wing coloration, and a moderately setose body (Fig. 4a–c). The apical posteroventral seta on the hind tibia matches the adjacent seta; the propleuron is bare; the upper calypter is larger than the lower calypter; and the cercus is elongated and oval. The onion flies (*Atherigona orientalis*) have a dark-brown to black antennae and palpi, pale grey pruinose thorax, sublateral black spots on the third, fourth, and fifth abdominal tergites (Fig. 4d–f). Their body length is about 4 mm with coarse reticulation ventral side and short leg bristles bare arista.

Sap beetles (*Urophorus humeralis*) have a broad head that is narrower than the pronotum (Fig. 4g–i). They have truncate elytra with three exposed chitinised dorsal segments and apically located yellow patches on both elytrons. They have moderately short club-shaped antennae and their mandibles with tooth-like structures on the inner side behind the apex. They have a broad lacinia bearded on the inner margin and transverse prothorax almost as wide as the elytra. The wireworms (*Agriotes* spp.) have straight body, 29 mm long, and are dark yellow in colour (Fig. 4j). They have nine abdominal segments that are visible dorsally, with the ninth segment terminating in a blunt point with two "eye spots". Their setae are centrally located on the dorsum of the 9^th^ segment, the 10^th^ segment bears the anus and lies ventrad to the 9^th^ segment. The frontoclypeal is lyre-shaped while their labium and maxillae are fused and the mandible bears tooth-like structures on the dorsal cutting edge.

The white grubs (*Maladera* spp.) have body length of 26 mm, 2.6 mm wide smooth yellow cranium and well-developed mesothorax legs (Fig. 4k). The apex of mandible and precoileae sutures are dark brown while the clypeus is subtrapezoidal with the anterior margin slightly shorter than the posterior margin. The labrum has a medial ridge, whereby the first to third abdominal segments are slightly thicker than the other abdominal segments and the teges of raster make up about half of the posterior half (Fig. 4k).

### Molecular characteristics of cabbage and onion flies

The onion fly samples were positively characterised using mitochondrial COI gene, and their sequences linked to publicly available *Atherigona orientalis* COI sequences with identity similarities of ≥ 99% (Table 2), while the cabbage fly samples linked to *Delia platura* with identity similarities of ≥ 97%. For the mitochondrial COI gene region, both GenBank (NCBI) and BOLD queries gave similar identities. The D2 region of 28S large subunit rDNA corroborated the characterisation achieved by the mitochondrial COI gene region, whereby the *Delia platura* linked with ≥ 99% similarity (Table 2). However, the D2 region of 28S rDNA could only resolve the onion pests up to genus level with ≥ 97.56% similarity. The *Atherigona orientalis* sequences generated in this study were submitted to GenBank and assigned accession numbers (OQ835541–OQ835545 and OQ832304–OQ832304) while *Delia platura* sequences were assigned GenBank accession numbers OQ835546–OQ835550 and OQ832299–OQ832303 (Table 2).

**Table 2:** Characterisation summary of onions and cabbage pest samples using the mitochondrial COI and 28S rDNA domain 2 (D2) gene regions.

Sample ID	ID from GenBank	Assigned GenBank accession	Gene region	Similarity (%) with GenBank accessions (COI)	Assigned GenBank accession	Gene region	Similarity (%) with GenBank accessions (28S rDNA)	Source	BOLD ID	BOLD ID%
DF1	*Atherigona orientalis*	OQ835541	COI	99.66	OQ832304	28S rDNA	97.56	Onions	*Atherigona orientalis*	100
DF2	*Atherigona orientalis*	OQ835542	COI	99.37	OQ832305	28S rDNA	97.2	Onions	*Atherigona orientalis*	100
DF3	*Atherigona orientalis*	OQ835543	COI	100	OQ832306	28S rDNA	97.57	Onions	*Atherigona orientalis*	100
DF4	*Atherigona orientalis*	OQ835544	COI	100	OQ832307	28S rDNA	97.02	Onions	*Atherigona orientalis*	100
DF5	*Atherigona orientalis*	OQ835545	COI	99.85	OQ832308	28S rDNA	97.55	Onions	*Atherigona orientalis*	100
CF1	*Delia platura*	OQ835546	COI	97.87	OQ832299	28S rDNA	99.53	Cabbage	*Delia platura*	97.94
CF2	*Delia platura*	OQ835547	COI	97.56	OQ832300	28S rDNA	99.77	Cabbage	*Delia platura*	97.94
CF3	*Delia platura*	OQ835548	COI	97.87	OQ832301	28S rDNA	99.09	Cabbage	*Delia platura*	97.94
CF4	*Delia platura*	OQ835549	COI	97.38	OQ832302	28S rDNA	100	Cabbage	*Delia platura*	97.94
CF5	*Delia platura*	OQ835550	COI	97.72	OQ832303	28S rDNA	99.54	Cabbage	*Delia platura*	97.94

### Management of insect pests

**Figure 5:**
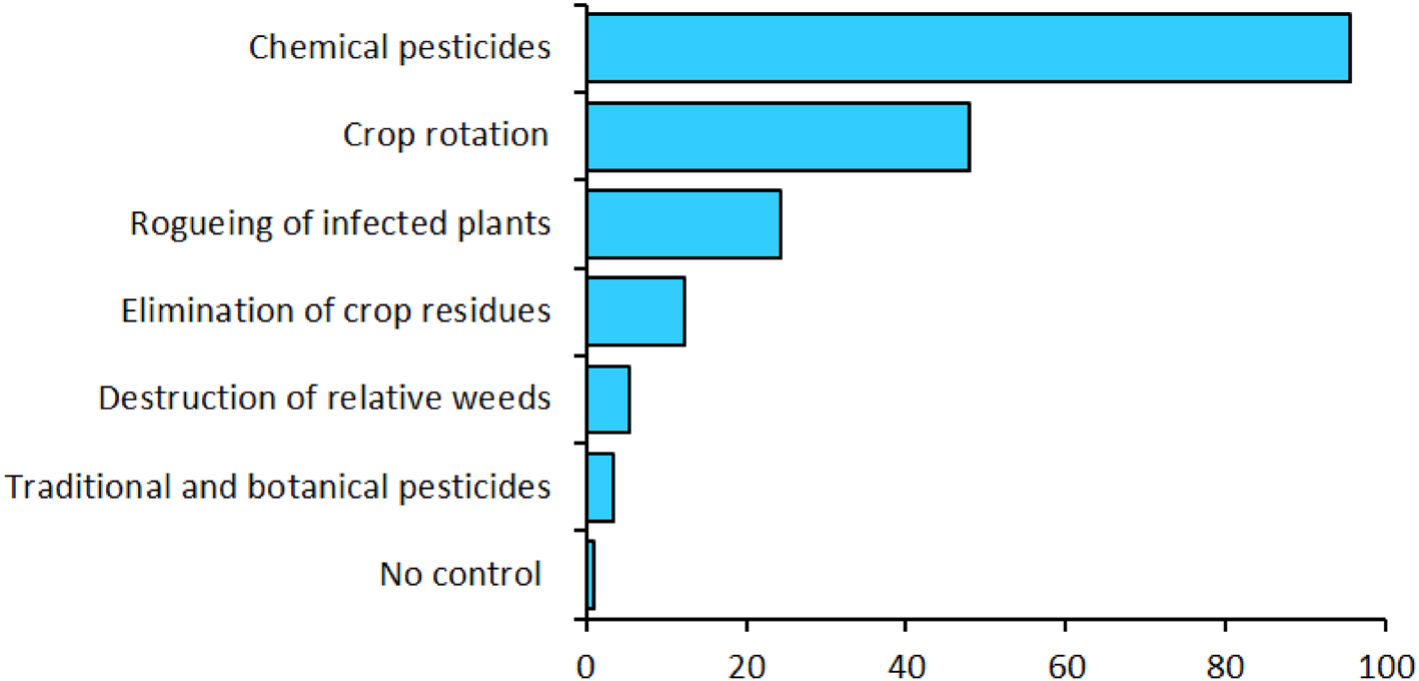
Pest management approaches used in major vegetable production areas of Kenya.

Almost all of cabbage and onion farmers in the study area relied on chemical pesticides to control insect pests (Fig. 5). About half of the farmers demonstrated knowledge and utilisation of crop rotation in the management of both insect pests and diseases of cabbage and onions. Other methods of insect pest management included rogueing, elimination of damaged crop residues, and destruction of weed hosts (Fig. 5). Generally, the application of botanical pesticides and traditional pest management approaches was limited. There was a weak negative correlation between pest management approaches and the incidence and severity of soil-dwelling pests except for the control of relative weeds, which showed no correlation (Table 3). There was a significant positive correlation between absence of pest control and the occurrence of soil-dwelling pests (Table 3).

**Table 3:** Correlation between pest management approaches and the incidence and damage severity of soil-borne pests.

Explanatory variables	Response variables	
	Pest incidence	Damage severity
Insecticides	− 0.25	− 0.31*
Crop rotation	− 0.24	− 0.19
Rogueing	− 0.10	− 0.16
Elimination of crop residues	0.25	− 0.03
Destruction of relative weeds	0.09	0.03
Traditional and botanical pesticides	− 0.04	− 0.08
No control	0.41**	0.36*

*Denotes statistical significance at *p* < 0.05.

**Figure 6:**
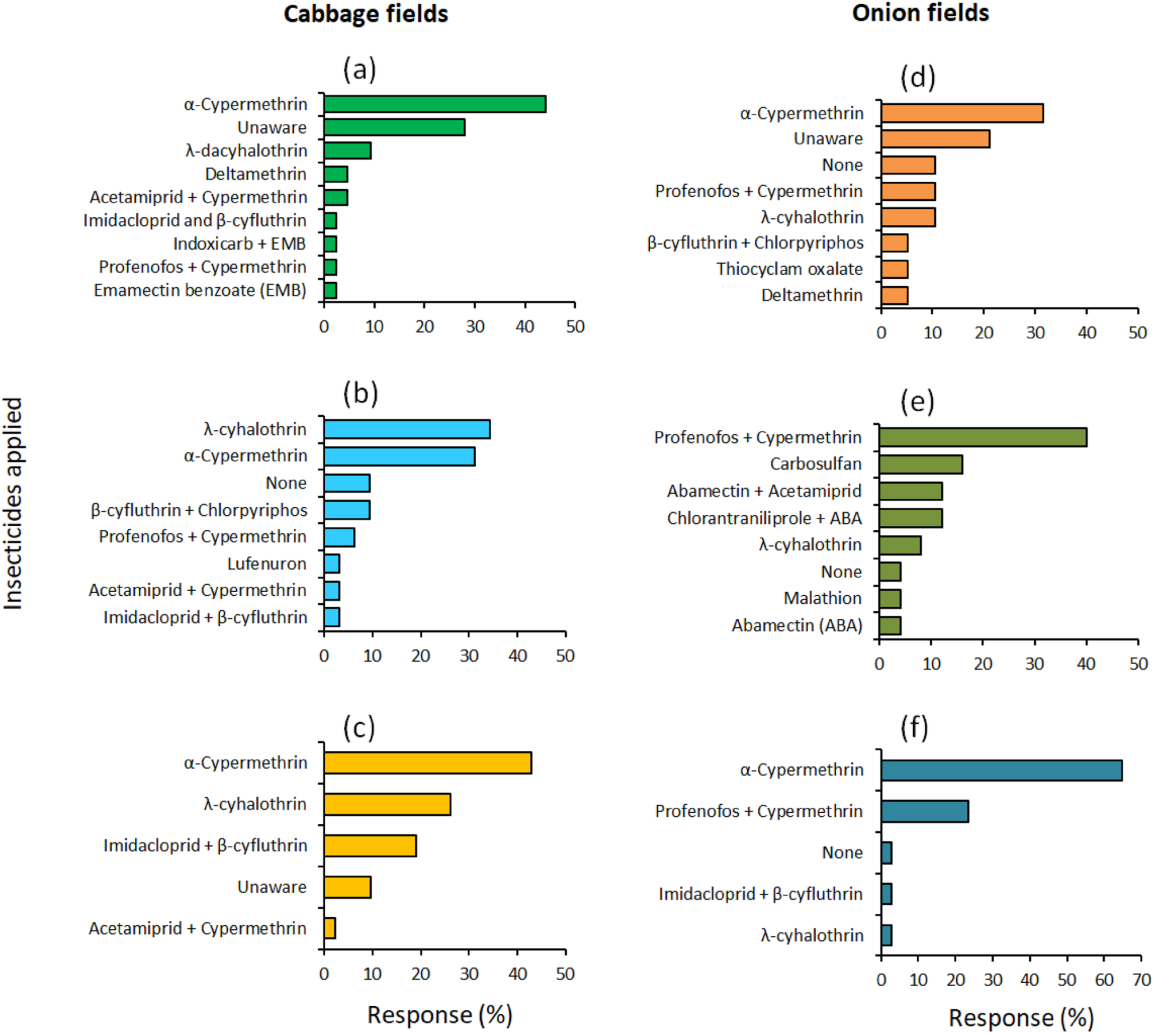
Insecticides used in cabbage (**a**–**c**) and onion fields (**d**–**f**) in Nakuru (**a** and **d**), Kiambu (**b**), Nyandarua (**c**), Kajiado (**e**), and Nyeri (**f**).

Alpha-cypermethrin, lambda-cyhalothrin, imidacloprid, beta-cyfluthrin, and acetamiprid were the most commonly applied chemical pesticides by cabbage farmers in Nyandarua, Nakuru, and Kiambu (Fig. 6). Pesticides with profenofos and cypermethrin were also common in Kiambu and Nakuru, while beta-cyfluthrin and chlorpyriphos were only used by farmers in Kiambu (Fig. 6). It was noted that a significant fraction of farmers in Nakuru and Nyandarua were unaware of the insecticides they used in pest management. The difference in pesticide use across the three cabbage-growing counties was statistically significant (χ2 = 54.87, df = 22, *p* < 0.001). Male farmers were more likely to use pesticides compared to their female counterparts, but the relationship between gender and pesticide use was not significant (χ2 = 9.92, df = 11, *p* = 0.537). Whereas pesticides were mostly used by farmers with primary and secondary school education, there was no significant relationship between the choice of pesticide and level of education (χ2 = 41.57, df = 22, *p* = 0.007).

In onion fields, insecticides with profenofos and cypermethrin as active components were the most used by farmers in Nakuru, Kajiado, and Nyeri, while alpha-cypermethrin was predominantly used Nyeri and Nakuru (Fig. 6). Carbosulfan, chlorantraniliprole, abamectin, and acetamiprid were commonly used in Kajiado (Fig. 6). A considerable fraction of farmers in Nakuru, Kajiado, and Nyeri did not apply any synthetic pesticides. In Nakuru, however, 1 in 5 farmers were unaware of the names of pesticides used in their farms (Fig. 6). There were significant differences in the pesticides used by onion farmers in the study area (χ2 = 71.46, df = 26, *p* < 0.001). However, the relationships between gender and education level, and the choice of pesticide used in onion fields were not significant (gender: χ2 = 13.50, df = 13, *p* = 0.41, education level: χ2 = 39.43, df = 39, *p* = 0.451).

### Frequency of pesticide use

**Figure 7:**
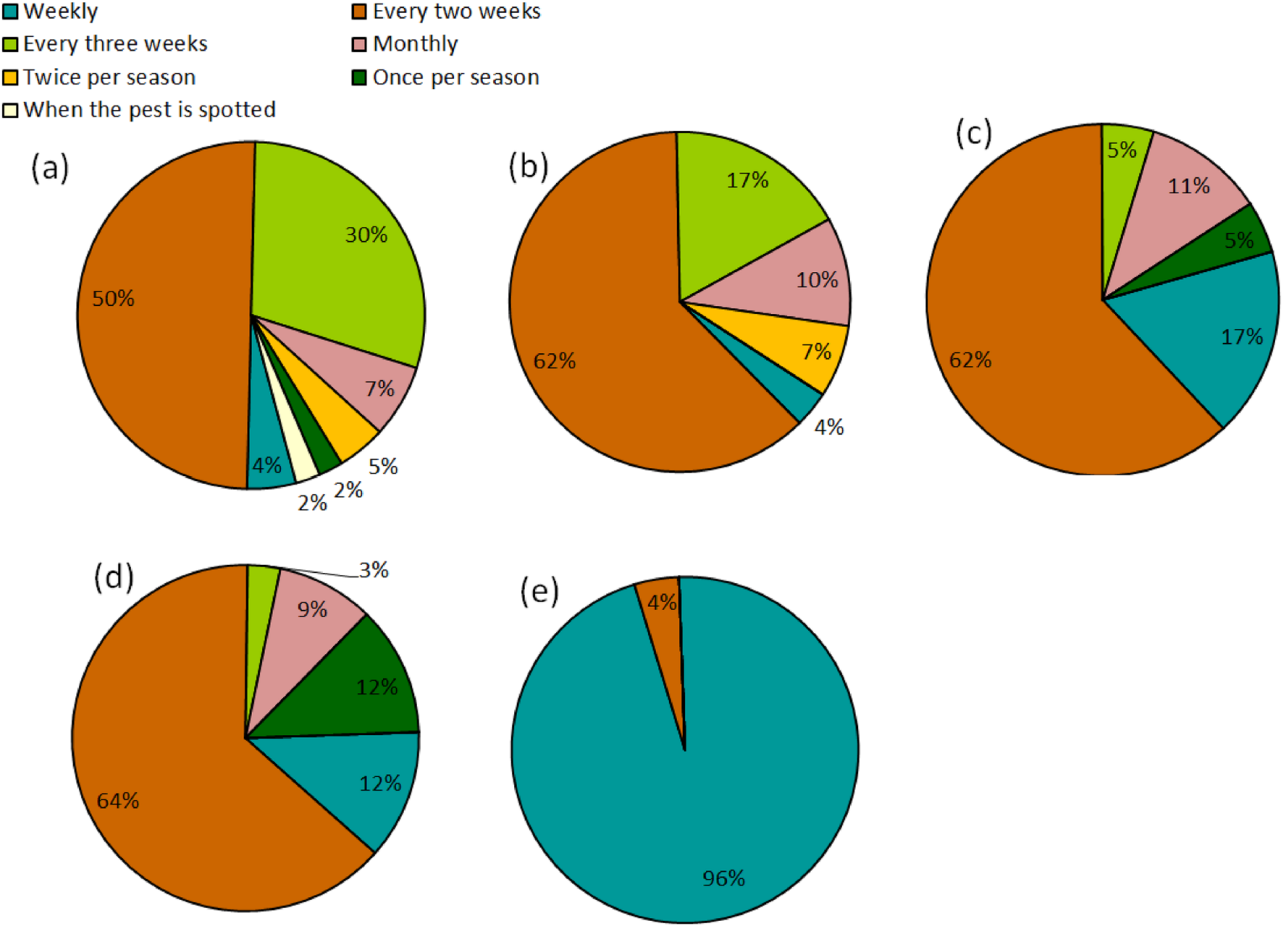
Frequency of pesticide application by cabbage and onion farmers in Nyandarua (**a**), Kiambu (**b**), Nakuru (**c**), Nyeri (**d**) and Kajiado (**e**).

The frequency of weekly application of insecticides was predominant in Kajiado, while bi-monthly application was most common in the other areas (Fig. 7). Although farmers with primary education were more likely to apply insecticides weekly, the level of education did not have a significant relationship with the frequency of pesticide application in cabbage (χ2 = 18.47, df = 12, *p* = 0.102) and onion fields (χ2 = 9.12, df = 12, *p* = 0.691). Agroecological zone had a significant influence on the frequency of pesticide application in onion fields (χ2 = 99.95, df = 20, *p* < 0.001) but not in cabbage farms (χ2 = 38.64, df = 30, *p* = 0.134). Nonetheless, there was no clear relationship between frequent pesticide application and incidence of soil-borne pests (*r* = − 0.05, *p* = 0.729).

## Discussion

### Occurrence, incidence and damage severity of soil-dwelling vegetable pests

The current study revealed high presence of soil-borne pests in vegetable farms across Kenya. The presence of several soil-borne pests, such as cabbage root fly (*Delia platura*) and onion root fly (*Atherigona orientalis*)*,* white grubs (*Maladera* sp.), wire worms (*Agriotes* sp.), and sap beetles (*Urophorus humeralis*) agrees with previous studies that have reported soil-borne insect pests are a major biotic challenge to crop production in Kenya and beyond^28,33,37–39^. The larval stage of *D. platura* is polyphagous foraging on crops from different families including Brassicaceae, Amaryllidaceae, Poaceae, Fabaceae Apiaceae and Cucurbitaceae, among others^40–45^. Previous observations have reported moderate to high damage by* Delia platura* Meigen, *Delia radicum* L. and *Delia antiqua* Meigen in cabbage and onion fields, especially in nearctic and palaearctic regions^22,46^ The presence of adult *D. platura* in cabbage fields with no signs of larval infestation could be the third generation flies which emerge when the crop is in physiological maturity, thus causing the least or uneconomical damage^47–49^. Furthermore, application of broad-spectrum pesticides and unfavourable climatic conditions, such as the dynamic temperatures might have reduced egg production, enhanced egg predation and caused insect starvation^48^.

The current study found high incidence of onion maggot with damage index scores of 4 (considerable damage and infestation) and 5 (very high infestation levels/wilted and dead plants) in lower and upper highland AEZs (UH2, LH3 and LH4). This aligns with previous cases reported in Pakistan, where infestation ranges from 25 to 85% in melon fruits^50^. *Atherigona orientalis* is a polyphagous pest that feeds on plant materials damaged by other pests, but it can also be a primary pest in Solanaceae vegetables. For instance, *A. orientalis* is a major pest of *Capsicum annuum* in Nigeria, causing severe damage to both ripe and unripe pepper fruits, whereas the pest has been found on tomatoes and garlic chives in Korea^51,52^. Whilst *A. orientali*s was first recorded in Kenya (Kilifi) in 1999 by Robert S. Copeland, this is the first report of the insect as a major host of onion in the country. *A. orientali*s has a broad distribution globally and has previously been reported in afrotropical, nearctic, neotropical, Indo-Malayan, and Australasian regions as a major pest of cauliflower and cabbage (*Brassica oleracea* L.)*,* orange (*Citrus sinensis* L.)*,* bell pepper (*Capsicum annuum* L.)*,* tomato (*Lycopersicon esculentum* Miller)*,* and melon (*Cucumis melo* L.)*, Sorghum bicolor* L. Moench and *Phaseolus* spp.^52–54^. The absence of onion root flies in lower midland zone (V) of Kenya can be attributed to the semi-arid agro-ecological conditions characterised by low altitude (970–1390 m), annual mean rainfall (420–520), and day temperature range of (23–28 °C), which may result in insect mortality due to desiccation^55^.

Other polyphagous pests, such as white grubs and wireworms were detected in three out of the five counties surveyed. Adult *Maladera* spp. can cause severe leaf damage to a broad range of horticultural crops, while the larvae feed on plant roots and cause substantial harm to horticulture and forestry^56,57^. The current study found a damage index score of 3 (average damage of 5–50%) and a high infestation of vegetables at the seedling stage, causing wilting and plant death. The current study did not detect any damage by the adult beetles, possibly due to their nocturnal feeding nature^57^. It should be noted that white grubs have been declared a biosecurity threat with a history of biological invasion^58^. The invasive species have also been reported in the United States, the Republic of Georgia, Turkey, Canada, and Rwanda^37,58–60^.

Previous studies have reported over 39 species from 21 genera of wireworms attacking potatoes, carrots, and sugar beets, especially in Europe and America^20,61–64^. While the damage intensity of wireworms on cruciferous crops has not been quantified in SSA, global studies have reported severe feeding damage on sweet potatoes^62,63,65–67^. Wireworms, soil-inhabiting larvae of click beetles (Coleoptera: Elateridae), are critical pests of arable and horticultural crops that feed on vegetable roots, causing retarded plant growth^68,69^. The consequent root damage hampers absorption of nutrients, leading to reddish or purple discoloration and the narrowing and curling of leaves^70^. Wireworms can cause 100% crop damage when infestation occurs during the seedling stage, leading to poor crop stands^64^. Studies in North America and Europe have identified numerous wireworm species with devastating effects on a broad range of crops^62,68,71^. However, the larvae of most sub-Saharan species remain undescribed, with no DNA sequence data.

Sap beetles or pineapple sap beetles (*Urophorus humeralis*) (Coleoptera: Nitidulidae) were detected in Kieni (upper Highland), causing severe rot and wilting (up to 100% crop damage) on mature onion bulbs. Similar results have been reported on the strawberry sap beetle, where crop damage on semi-ripe and ripe fruits was 100%, while unripe fruits showed over 82% damage following 48 h of exposure^72^. The pest was primarily detected alongside onion maggots, acting as a secondary pest of field onions in some cases. *U. humeralis* was also associated with fusarium basal rot of onions (*Fusarium oxysporum* Schlecht), indicating that the pest either attacks fermenting onions due to fusarium basal rot or is involved in the transmission of the disease. This observation aligns with Konam and Guest^73^, who found that Scolytidae and Nitidulidae beetles were attracted to *Phytophthora palmivora* disease lesions and facilitated disease transmission. Moreover, sap beetles are attracted to volatile compounds produced by *Fusarium verticillioides* in maize^73–75^, highlighting the need to establish the interaction between *U. humeralis* and onion crops infested with onion maggots and *Fusarium* basal rot.

The study demonstrated that farmers’ education level and awareness of soil-borne pests has an integral effect on the incidence of soil-dwelling pests and crop production. These results conform to previous findings, which reported a relationship between farmers’ education and the choice of crop variety, time of planting, crop husbandry, pest management practices, technology adoption, crop productivity^76–81^. Nevertheless, the high preference for hybrid vegetable varieties could be associated with their tolerance to pests. Moreover, previous studies have demonstrated substantial inclination towards improved vegetable varieties due to qualities such as high yield, compactness, early maturity, disease resistance, prolonged shelf life, and strong pungency for onions^82–84^.

### Management of soil-dwelling pests

The current study revealed that almost all vegetable farmers in the study area rely on chemical pesticides to control pests. This is in agreement with previous studies, which revealed that 70–95% of Kenyan farmers use synthetic pesticides for the management of vegetable and fruit pests^85,86^. The absence of soil-borne onion insect pests in Oloitokitok could be largely attributed to the high frequency of pesticide application observed during the study, whereby most farmers applied pesticides weekly and in excess doses. The high frequency of application in excessive dosage has been previously reported^87,88^ and could be due to the ease of access to chemical pesticides at a low cost from neighbouring Tanzania. It was noted that some of the commonly used pesticides, such as pyrethroids (lambda-cyhalothrin, alpha-cypermethrin, and beta-cyfluthrin) and neonicotinoids (imidacloprid), are moderately hazardous to human health and the environment, according to the WHO classification (WHO II) (Table 4)^89^. Moreover, a quarter of the farmers interviewed were unaware of the specific product names of the pesticides used on their farms. Inadequate knowledge on pesticide use greatly contributes to excessive application, which could lead to pesticide toxicity, pest resistance, and ecosystem damage.

**Table 4:** Characterisation of chemical pesticides used by farmers in major vegetable growing areas of Kenya. *Source* Pest Control Products Board of Kenya^89^.

Active component	Product names	WHO class	Chemical class	Mode of action
Lambdacyhalothrin	Duduthrin 1.75 EC, Halothrin 2.5 EC, Karate 2.5 WG, Pentagon 50 EC, Vendex 50 EC	WHO Class II (Moderately hazardous)	Pyrethroid	Contact, ingestion and ovicidal action
Imidacloprid and beta-cyfluthrin	Thunder 145 OD, Buffalo 100 OD	WHO Class II (Moderately hazardous)	Imidacloprid is a neonicotinoid while beta-cyfluthrin is a pyrethroid	Contact and systemic residual action
Beta-cyfluthrin + Chlorpyriphos	Betaforce 263 EC	WHO Class II (Moderately hazardous)	Beta-cyfluthrin is a pyrethroid while Chlorpyriphos is an organophosphate pesticide	Contact, stomach and respiratory action
Alpha-Cypermethrin	Bestox 20 EC, Tata Alpha 10 EC	WHO Class II (Moderately hazardous)	Pyrethroid	Disruption of Voltage-gated sodium channel (VGSC) function
Acetamiprid + Cypermethrin	Aster Extrim 20 SL, Twiga Ace 20 SL	WHO Class II (Moderately hazardous)	Acetamiprid is a chloropyridinyl neonicotinoids while Cypermethrin is a pyrethroid	Systemic, translaminar action (acetamiprid), contact & stomach action (cypermethrin)
Thiocyclam hydrogen oxalate	Taurus 500SP	WHO Class II (Moderately hazardous)	Nereistoxin analogue	Contact and stomach actions
Deltamethrin	Decis 2.5 EC, Decis Forte EC 100EC	WHO Class II (Moderately hazardous)	Pyrethroid ester insecticide	Ingestion and direct contact
Emamectin benzoate	ESCORT 19 EC	WHO Class III (Moderately hazardous)	Avermectin	Disruption of the nerve impulses
Profenofos + Cypermethrin	Profile 440 EC, Profecron 44 EC,	WHO Class II (Moderately hazardous)	Profenofos is an organophosphate insecticide while Cypermethrin is pyrethroid	Acetyl cholinesterase (AChE) inhibitor with contact and stomach action
Indoxicarb + Emamectin Benzoate	Benocarb 100SC	WHO Class II (Moderately hazardous)	Indoxacarb is an oxadiazine pesticide while Emamectin Benzoate is an avermectin	Indoxicarb acts by blocking the neuronal sodium channels, while Emamectin Benzoate disrupts the nerve impulse
Lufenuron	Match 050 EC	WHO Class III (Moderately hazardous)	Imidacloprid is aneonecotinoid Insect Growth Regulators	Inhibits chitin synthesis and interfere with moulting
Chlorantraniliprole + Abamectin	Voliam Targo 063SC	WHO Class II (Moderately hazardous)	Chlorantraniliprole is an anthranilamide insecticide while Abamectin is an avermectin	Chlorantraniliprole is a ryanodine receptor modulator while Abamectin is a GABA agonist
Carbosulfan	Marshal 250 EC	WHO Class II (Moderately hazardous)	Carbamate insecticide	contact and stomach poison action
Abamectin	Acoster 5 EC	WHO Class II (Moderately hazardous)	Avermectin insecticide	Stimulates the gamma-aminobutyric acid (GABA) system
Malathion	Oshothion 50 EC	WHO Class II (Moderately hazardous)	Organophosphate insecticide	acetylcholinesterase inhibitor
Abamectin + Acetamiprid	Dudu Acelamectin 5% EC	WHO Class II (Moderately hazardous)	Abamectin belongs to avermectin class while acetamiprid is a chloropyridinyl neonicotinoids	Abamectin is a GABA agonist while acetamiprid has systemic and translaminar action

Past studies have reported high usage of pyrethroids and organophosphates in controlling insect pests in Kenya^86,90–92^. Although chemical application, crop rotation and rogueing enhanced pest suppression, the impact was not significant when applied in isolation. The low efficacy of insecticides draw attention to the possibility of pesticide resistance by soil-borne pests of cabbage and onions. Soil-borne pests, especially the root maggots have demonstrated a remarkable capacity to resist against organic pesticides, especially when applied in excess^14^. Poor pesticide use characterized by excessive dosage and short frequencies of application could lead to pesticide resistance. For example, farmers in Kajiado County expressed concern over above ground pests such as *Thrips tabaci*. The poor pesticide use observed in Kajiado has been previously reported^93–95^, and could also be attributed to low literacy levels; whereby, about three-quarters of farmers in the area had a primary school education or below. Therefore, there is a need to create awareness and build capacity among farmers on the effective use of pesticides for sustainable pest management. The absence of soil dwelling pests in Kajiado is probably due to basin irrigation, which might have resulted in drowning and/or suffocation, and the high frequency of insecticide application.

Almost half of the farmers used crop rotation as an additional approach to pest management. Non-chemical approaches to pest management, such as rouging of infected crops, destruction of relative weeds, use of traditional and botanical pesticides, and elimination of crop residues, are essential in the integrated management of insect pests, although farmers did not leverage these approaches^96^. Therefore, there is a need to promote integrated pest management approaches among vegetable farmers in Kenya for the effective management of insect pests as well as crop diseases.

## Conclusion

The current study detected the presence of soil-dwelling insect pests with devastating effects on vegetable farming in Kenya. *Delia platura* and *Antherigona orientalis* are invasive polyphagous species with moderate to high occurrence in lower and upper highlands.

This is the first report of *Antherigona orientalis* as a major pest of onions and *Delia platura* as a potential pest of cabbage in Kenya. Other soil-borne pests identified across different agroecological zones include white grubs, wireworms, and sap beetles. The severity of the pest damage was high, especially in fields affected by *Delia platura*, *Antherigona orientalis* and *Urophorus humeralis*. It was noted that soil-dwelling pests which occurred in association, such as sap beetles and onion maggots aided in the transmission of plant diseases, especially Fusarium basal rot, causing further yield losses. Majority of the farmers apply broad-spectrum insecticides, primarily pyrethroids and organophosphates, on a weekly or fortnightly basis, but with little success. Although one in every two farmers used crop rotation to manage pests, integrated pest management approaches have not been embraced in vegetable cropping systems. The multi-infestation witnessed in onion production systems manifests the impact of climate change on crop production, whereby insect pests have expanded their host domain to survive. Moreover, the impacts of soil-dwelling pests have been exacerbated by a lack of accurate information and poor pesticide use, arising from the limited awareness and low education levels of most farmers. Thus, there is a need for rigorous classification of the prevailing soil-dwelling species, their phenology, and polyphagy in the context of tropical climate to design effective integrated pest management approaches.

## Materials and methods

### Characteristics of the study area

**Figure 8:**
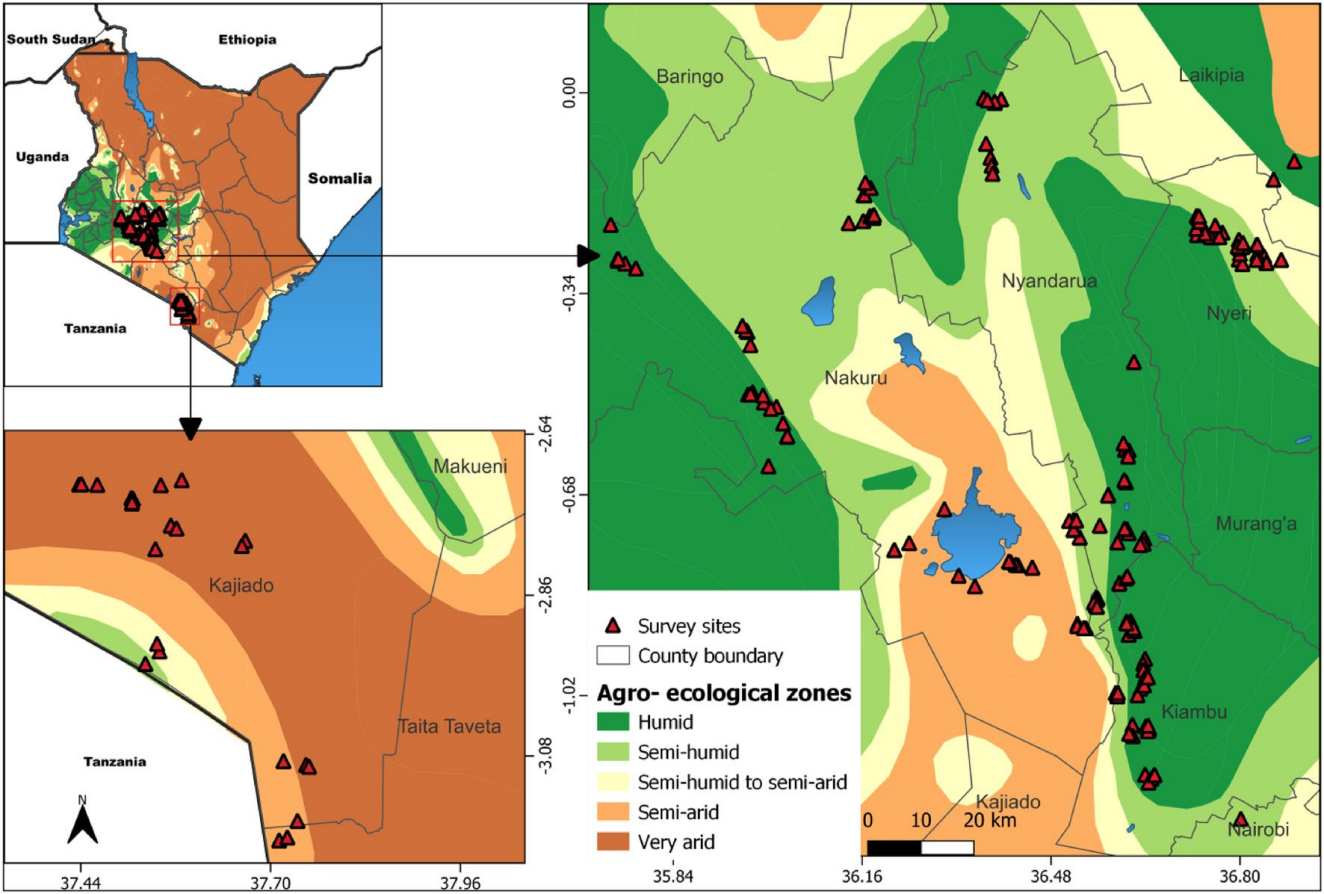
Survey sites and agro-ecological zones considered during the study.

A field survey was conducted for three months (April to June 2022) to determine the occurrence, incidence, and severity of soil-borne pests of cabbage and onions in the major growing zones of Kenya: Nyandarua, Nakuru, Kiambu, Kajiado, and Nyeri counties (Fig. 8). The counties were selected to represent the major agroecological zones where cabbage and onions are grown: high altitude (Nyandarua, Nakuru, and Kiambu) and middle altitude (Nyeri and Kajiado) (Table 5). A total of 210 fields were surveyed during the study: 45, 65, 32, 34, and 34 fields in Nyandarua, Nakuru, Kiambu, Kajiado, and Nyeri, respectively. Apart from Nyandarua which had more female farmers, the other counties were dominated by male farmers. Most farmers were aged between 45 and 54 years and had attained at least a primary school education. Almost all farmers interviewed grew onions and cabbage on small land pieces of ≤ 1 acre, except in Kajiado County where nearly half of the farmers planted the vegetables on 2–4 acres (Table 6). Gloria F1, Pructor F1, and Victoria F1 were the three most common cabbage varieties in cabbage-growing counties, whereas Russet F1, Mang’ola, Malbec F1, and Red Coach F1 were the most grown bulb onion varieties in onion-growing areas (Table 7).

**Table 5:** Characteristics of the counties covered in the survey of soil-borne insect pests. *Source* Jaetzold and Schmidt^55^.

County	Sub-county	Agroecological zones AEZ	Target crop	Altitude (m.a.s.l)	Mean temperature (°C)	Mean annual rainfall (mm)
Nakuru	Molo, Bahati, & Njoro	Upper highland (UH1)	Cabbage	2400–3000	10.0–14.6	1150–1600
	Molo South	Upper highland (UH2)	Cabbage	2400–3000	10.0–14.6	1000–1200
	Naivasha & Njoro	Lower highland (LH3)	Cabbage & Onion	2250–2280	15.0–15.2	800–900
	Naivasha	Lower highland (LH4)	Onion	2190–2280	15.0–15.6	800–900
Kiambu	Lari & Kijabe	Upper highland (UH1)	Cabbage	2400–3000	10.0–14.6	1150–1600
	Kijabe	Upper highland (UH2)	Cabbage	2400–3000	10.0–14.6	1000–1200
Nyandarua	South Kinangop	Upper highland (UH1)	Cabbage	2400–3000	10.0–14.6	1150–1600
	North Kinangop	Upper highland (UH2)	Cabbage	2400–3000	10.0–14.6	1000–1200
	Ol Kalau, & Ol Joro Orok	Upper highland (UH3)	Cabbage	2370–2430	13.7–14.7	900–1100
Nyeri	Kieni	Upper highland (UH3)	Onion	2370–2430	13.7–14.7	900–1100
Kajiado	Oloitokitok	Lower midland (LM5)	Onion	970–1 390	20.1–22.7	420–520

**Table 6:** Demographic characteristics of the respondents in the study area.

Variables		Nyandarua	Nakuru	Kiambu	Kajiado	Nyeri	Overall
		(%)					
Gender	Male	44.4	58.5	56.3	88.0	67.7	60.2
	Female	55.6	41.5	43.8	12.0	32.4	39.8
Age of the farmer (years)	18–24	2.2	3.2	3.1	4.0	0.0	2.5
	25–34	4.4	6.4	3.1	12.0	5.9	6.0
	35–44	22.2	17.5	25.0	20.0	23.5	21.1
	45–54	35.6	38.1	31.3	44.0	52.9	39.7
	55–64	26.7	22.2	18.8	16.0	14.7	20.6
	≥ 65	8.9	12.7	18.8	4.0	2.9	10.1
Education level	None	0.0	0.0	0.0	8.0	2.9	1.5
	Primary	44.2	50.0	35.5	64.0	50.0	48.2
	Secondary	39.5	35.5	32.3	24.0	38.2	34.8
	Tertiary	16.3	14.5	32.3	4.0	8.8	15.4
Land area dedicated to crop production (acres)	≤ 1	77.8	72.3	78.1	20.0	91.2	71.1
	2–4	20.0	24.6	21.9	44.0	8.8	22.9
	5–7	2.2	0.0	0.0	24.0	0.0	3.5
	≥ 8	0.0	3.1	0.0	12.0	0.0	2.5

**Table 7:** Cabbage and onion varieties grown by farmers in the study area.

Varieties	Cabbage growing counties (%)				Varieties	Onion growing counties (%)			
	Nyandarua	Nakuru	Kiambu	Total		Nyeri	Nakuru	Kajiado	Total
Gloria F1	38.1	40.5	21.9	34.5	Russet F1	44.1	0.0	0.0	18.5
Pructor F1	19.1	16.7	46.9	25.9	Mang’ola	0.0	0.0	60.0	18.5
Victoria F1	14.3	23.8	9.4	16.4	Malbec F1	14.7	35.0	0.0	14.8
Copenhagen	11.9	0.0	6.25	6.0	Red Coach F1	0.0	0.0	36.0	11.1
Kilimo F1	9.5	0.0	3.1	4.3	Red Creole	0.0	30.0	0.0	7.4
Greenboy F1	0.0	9.5	0.0	3.5	Jambar F1	14.7	0.0	0.0	7.4
Globemaster F1	2.4	2.4	0.0	1.7	Super Yali	8.8	0.0	0.0	3.7
Rossy F1	0.0	0.0	6.3	1.7	SV7030NS F1	8.8	0.0	0.0	3.7
Fabiola F1	0.0	0.0	6.3	1.7	Matahari F1	0.0	15.0	0.0	3.7
Tropicana	0.0	2.38	0.0	0.9	Texas Grano F1	0.0	5.0	3.0	3.7
Baraka F1	0.0	2.38	0.0	0.9	Africa Red F1	0.0	10.0	0.0	2.5
Kiboko f1	0.0	2.38	0.0	0.9	Red Shine F1	0.0	0.0	4.0	2.5
Zawadi F1	2.38	0.0	0.0	0.9	Red King F1	3.0	0.0	0.0	2.5

### Data collection

In each county, the fields were selected under the county agricultural extension officers' guidance based on the availability of the target crop. Figure 8 presents the farms that were considered during the study. Plant sampling was conducted using a zigzag pattern in each crop field; plants inside the field and those along the edges were targeted based on the feeding behavior of the target pest. In each field, fifty plants were randomly sampled and assessed for symptoms of subterranean pest infestation such as wilting, stunting, discolouration of leaves and stems, loss of vitality and tissue distortion^48,97^. Field assessments were scheduled to coincide with the period when cabbage and onion plants were at seedling, vegetative and early maturity stages. This is because the damage caused by soil-borne pests, is exceptionally severe during the early stages of growth stages^48^.

Plants showing these symptoms and some apparently healthy plants were uprooted and assessed for signs of infestation, such as the presence of actively feeding larvae, plenitude of insect frass and eggs near the crown, and abundance of pupae^48,97^. Plants harbouring soil-borne insects were counted, and the percentage incidence was determined using Eq. (1). The collection of insect and plant samples was carried out in compliance with the guidelines provided by Kenya’s National Council for Science Technology and Innovation (NACOSTI) and Kenya Plant Health Inspectorate Services (KEPHIS).

1 $$Pest \;incidence \left(\%\right)=\frac{\mathrm{Number\; of\; infested\; plants}}{\mathrm{Total\; plants\; observed }} \times 100$$

To assess the infestation severity of the soil-borne pests on each plant, five different categories (a scoring scale) were created based on the intensity of the infestation and crop damage: whereby a score of 1 = absence of damage and infestation, 2 = slight infestation (< 5% plant parts damaged), 3 = average infestation (> 5 and < 50% plant parts damaged), 4 = considerable infestation (> 50% plant parts damaged) with severe stunting or wilting, and 5 = very high infestation levels and severe damage or wilted and dead plants^98^. The infestation severity index for each field was determined using Eq. (2)^99^.

2 $$Infestation\; severity \left(\%\right)=\frac{\mathrm{Sum\; total \;score\; of \;the\; infested\; plants}}{\mathrm{Total\; number \;of \;infested \;plants\; observed }}\times 100$$

Plants with signs of infestation were extracted intact with the soil adhering to the roots, placed in a four-litre container, labelled, and transported to the International Center of Insect Physiology and Ecology (*icipe*) research laboratory, Nairobi, Kenya, for further identification. The GPS coordinates indicating the locations of the sampled fields were recorded using a GPS machine (Garmin eTrex 20 × GPS).

### Assessment of demographic characteristics and crop production practices

A stratified sampling approach was employed to select farmers, whereby a stratum consisted of active cabbage and onion farmers. The sample size for each county was determined using a methodology developed by Sseruwagi et al.^100^. A purposive sampling technique was used to identify farmers who cultivated either cabbage or onions in the study areas. A closed-ended questionnaire was used to collect data on farmers’ demographic characteristics, agro-climatic data, crop production practices, pest occurrence, incidence and severity and management.

### Morphological identification of insects

The click beetles larvae were identified morphologically by examining the following set of characteristics defined by Glen and colleagues^101^. The body is straight with nine abdominal segments visible dorsally, the ninth segment terminating in a blunt point, and the tenth segment lying ventrad to the ninth with or without hooks. The larvae have three pairs of well-developed, sub-equal thoracic legs; a lyre-shaped frontoclypeal, labrium fused with the anterior margin of the frons and clypeus to form a rigid nasale. They have fused labium and maxillae and biforous spiracles.

Scarab beetles whose larval stage is called white grubs were identified using the morphological characteristic key by Šípek and Ahrens^21^. The beetles are 15.5–22 mm in length with smooth yellow cranium of 1.85–2.6 mm width. The apex and preciliae of the mandible are brown to black, with a white preclypeus and lyriform frontal sutures. They have a subtrapezoidal clypeus with a slightly shorter anterior margin than posterior margin and a weakly sclerotized preclypeus. The labrum has two pairs of prominent setae in the posterior half, trilobed anteriorly, and lateral lobes subdivided into mini lobes. The insect has antennomere with a large sensory spot and two smaller ventral spots, as well as a small spot, bearing minute setae at the apex. The raster teges cover at least the distal half of the ventral surface of the last abdominal segment.

Sap beetles were identified using the morphological characteristic key by Gillogly^102^. The body is elongated with at least three exposed chitinized dorsal segments. The labrum is bilobed with a tooth on the inner side behind the apex, and ligula has wide laterally protruding paraglossae. The insects have a stout palpi with a truncate and thickened terminal segment; transverse mentum, emerging in front; larger scutellum. The antennae are short, compact, and round in outline. The prosternal process behind the coxae is wide, round, and depressed, reaching the mesosternum, while the prothorax is narrower than the elytra.

Onion flies were identified using the keys and illustrations by Bohart and Gressitt^103^ and Grzywacz and Pape^104^. The flies have dark brown to black palpi and antennae and pale grey pruinose thorax with a pair of sublateral black spots on the 3rd, 4th, and 5th abdominal tergites. The insects are about 3.8 mm in length with a bare arista and coarse reticulation on the ventral surface. Morphological keys by Savage et al.^41^ and Darvas and Szappanos^105^ were used to identify cabbage root flies. They have a greyish body that is 5–9 mm long, and are moderately setose without body or wing colouration. The A1 vein extends to wing margin while the upper calypter is smaller or larger than lower one. The arista’s longest hair is shorter than the first flagellomere width. The insects have a bare propleuron and the hind tibia’s apical posteroventral seta is almost similar to the adjacent setulae or absent. In addition to morphological characterisation, the insect specimens were subjected to molecular characterization.

### Molecular characterisation

#### Sampling, DNA extraction and amplification

Insect pests from onion and cabbages were collected, preserved in absolute ethanol then brought to the Arthropod Pathology Unit of *icipe*, Nairobi, Kenya, for processing, whereby genomic DNA was extracted from individual insects using the Isolate II Genomic DNA Kit (Bioline, Meridian Bioscience, London, United Kingdom), following the the manufacturer’s instructions. The resultant DNA was eluted in a final 50 μL volume then quality and quantity checks done using the Nanodrop 2000/2000c Spectrophotometer (Thermo Fischer Scientific, Wilmington, USA). For characterisation, the mitochondrial COI gene region was targeted using LepF1 5’ ATTCAACCAATCATAAAGATATTGG 3’ and LepR1 5’ TAAACTTCTGGATGTCCAAAAAATCA 3’ markers^106^ in addition to amplification of the Domain 2 (D2) region of 28S large subunit rDNA using LepD2 Fw 5’ AGTCGTGTTGCTTGATAGTGCAG 3’ and LepD2 Rev 5’ TTGGTCCGTGTTTCAAGACGGG 3’ markers^107,108^. The PCRs were carried out in a total reaction volume of 20 µL containing 5X My *Taq* Reaction Buffer (5 mM dNTPs, 15 mM MgCl_2,_ stabilizers and enhancers), 0.5 pmol µl^−1^ of each primer, 0.5 mM MgCl_2_, 0.0625 U µl^−1^My *Taq* DNA polymerase (Bioline) and 15 ng µl^−1^ of DNA template. These reactions were set up in the Eppendorf Mastercycler^®^ Nexus Gradient Thermal Cycler (Eppendorf, Hamburg, Germany). The following cycling conditions were used: initial denaturation for 2 min at 95 °C, followed by 40 cycles of 30 s. at 95 °C, 30 s. annealing (52 °C for LepF1/R1 and 58.8 °C for LepD2 Fw/Rev) and 1 min at 72 °C, then a final elongation step of 10 min at 72 °C. The amplicons were resolved through a 1.2% agarose gel, then bands on the gel visualized and documented using the KETA GL imaging system trans-illuminator (Wealtec Corp, Meadowvale Way Sparks, Nevada, USA). Thereafter, the bands were excised and purified using Isolate II PCR and Gel Kit (Bioline) following the manufacturer’s instructions then shipped to Macrogen Europe BV (Meibergreef, Amsterdam, the Netherlands), for bi-directional sequencing.

#### Sequence analyses

The sample sequences were assembled and edited using Geneious Version 8 (http://www.geneious.com)^109^. The primer sequences were identified and removed from the consensus sequences generated (from both the forward and reverse reads). For conclusive identification of the species from both markers, similarity searches were conducted by querying the consensus sequences via BLASTn (Basic Local Alignment Search Tool) algorithm at the GenBank database hosted by National Centre of Biotechnology Information (NCBI). This algorithm aligns and compares the queried consensus sequences and the reference sequences deposited in the GenBank database. In addition to this, query was also done in BOLD (Barcode of Life Database).

### Data analysis

The percentage incidence and severity data were transformed using an arcsine transformation to satisfy the assumptions of homogeneity of variance and normality before analysis^110,111^. The data were subjected to IBM^®^ Statistical Package for Social Sciences (SPSS) 26 to generate descriptive statistics on demographic characteristics, crop varieties, pest incidence, pest severity, and management practices^112^. Chi-square tests (at the 5% significance level) were used to determine the relationships among the different variables. The data on percentage incidence and severity were subjected to an analysis of variance using R statistical software. A nonparametric correlation was conducted in SPSS to determine the relationship between pest incidence and the frequency of insecticide application.

## Data Availability

The datasets generated and/or analysed during the current study are available in the GenBank and accession numbers have been provided.
